# The Fecal Microbiota Transplantation: A Remarkable Clinical Therapy for Slow Transit Constipation in Future

**DOI:** 10.3389/fcimb.2021.732474

**Published:** 2021-10-22

**Authors:** Jiafei Liu, Liqiang Gu, Mingqing Zhang, Shiwu Zhang, Min Wang, Yu Long, Xipeng Zhang

**Affiliations:** ^1^ Department of Colorectal Surgery, Tianjin Union Medical Center, Nankai University, Tianjin, China; ^2^ Department of Pathology, Tianjin Union Medical Center, Nankai University, Tianjin, China; ^3^ Key Laboratory of Molecular Microbiology and Technology, Ministry of Education, TEDA Institute of Biological Sciences and Biotechnology, Nankai University, Tianjin, China

**Keywords:** slow transit constipation, fecal microbiota transplantation, intestinal neuron, short-chain fatty acids, intestinal motility

## Abstract

Slow transit constipation is a common condition that would be difficult to treat in clinical practice with a widespread incidence in the population. Pharmacotherapy and surgery are common treatment modalities. However, the clinical effect is limited, and patients still suffer from it. As the researchers strived in this field for decades, the profound relationship between slow transit constipation and fecal microbiota transplantation has comprehensively been sustained. It is very pivotal to maintain intestinal homeostasis, the structure function and metabolic function of symbiotic bacteria, which can inhibit the engraftment of intestinal pathogens. This mini review explains the treatment effects and possible mechanisms of the fecal microbiota transplantation in treating slow transit constipation. Simultaneously, it is found that there is significant improvement in the disease by adjusting the intestinal microbes like fecal microbiota transplantation. Fecal microbiota transplantation has efficient therapeutic effects in slow transit constipation compared with traditional therapies.

## Introduction

Fecal microbiota transplantation (FMT), as the major treatment of an untargeted microbiome modulation, means transplanting the functional flora from the feces of healthy people into the gastrointestinal tract of patients so as to reconstruct the new intestinal flora and realize the treatment of intestinal and extra-intestinal diseases (Gu et al., 2017). The aim of FMT is to correct dysbiosis by implanting feces collected from donors and treat the underlying disease (Matsuoka, 2020). The abundance and composition of intestinal flora are associated with a variety of diseases, and improving the abundance and composition of intestinal flora using FMT is a potential treatment for many diseases such as gastrointestinal, metabolic, nervous system, and autoimmune diseases. Ning Li et al. suggested that FMT was effective and safe for slow transit constipation (STC) (Ding et al., 2018). At short- and long-term follow-up, the Wexner constipation scale, stool consistency, and constipation symptoms got a huge improvement. This demonstrated that FMT combined with soluble dietary fiber (pectin) had efficacy in treating STC both in short term and long term (Zhang et al., 2018). In Ning Li et al.’s clinical trial, the FMT group had a 30% higher cure rate for treatment of STC than conventional treatment (Tian et al., 2017). FMT has been recommended for the treatment of recurrent or refractory *Clostridium difficile* infection (CDI) by many clinical guidelines and consensus. From Jessica R et al., FMT failed only in 5 of the 49 patients treated, while 45 patients underwent *Clostridium difficile* decolonization 1 week after FMT, and polymerase chain reaction was negative (Allegretti et al., 2020). In addition, FMT has shown certain efficacy in clinical studies on other diseases such as inflammatory bowel disease (IBD), irritable bowel syndrome (IBS) and functional constipation, and cirrhosis (McDonald et al., 2018; Mullish et al., 2018).

## The Intestinal Microflora in Human Gut

Researchers found that Actinobacteria, Bacteroidetes, Firmicutes, and Proteobacteria phyla are predominant in the human gut, while Bacteroidetes phyla, Firmicutes, and Proteobacteria are regularly found in the colon tract (Meng et al., 2018). In the human gut, it is the anaerobic bacteria that play a vital role throughout. Pivotal anaerobic bacteria species in the gut include the following: Firmicutes, Bacteroidetes, and Verrucomicrobia (Dupont et al., 2020). Alteration in the proportion of anaerobic bacteria frequently caused chronic disorders associated with dysbiosis. Proteobacteria in excess of 10% caused intestinal flora abnormalities and disorders (Shin et al., 2015). Above all, there are four pivotal anaerobic bacteria (Proteobacteria, Bacteroidetes, Firmicutes, and Verrucomicrobia) in the human gut connected with intestinal diseases. (1) Proteobacteria is one of the largest phyla of bacteria, including many pathogenic bacteria, such as *Escherichia coli*, *Salmonella*, *Vibrio cholerae*, *Helicobacter pylori*, and other well-known species (Shin et al., 2015). Microbial maladaptation of the intestinal microbiome is identified by Proteobacteria. (2) Bacteroidetes are the largest phylum of gram-negative bacteria inhabiting in the human gut, considered the leading players of the healthy state and sophisticated homeostasis safeguarded by gut microbiota (Gibiino et al., 2018). From the healthy FMT donors, Reetta et al. isolated intestinal commensal bacteria with anti-inflammatory capacity. They isolated Bacteroides and Parabacteroides, which were recognized as *Parabacteroides distasonis*, *Bacteroides caccae*, *Bacteroides intestinalis*, *Bacteroides uniformis*, *Bacteroides fragilis*, *Bacteroides vulgatus*, and *Bacteroides ovatus* through the whole genome sequencing (Hiippala and Kainulainen, 2020). (3) The Firmicutes/Bacteroidetes ratio was a possible biomarker of gut dysbiosis. In healthy adult humans, the intestinal bacteria Firmicutes were in the range of quantitative data of 20.5% up to 80%, while Bacteroidetes were from 13.85% up to 75.3%. Many studies found that, in humans, many diseases such as obesity were associated with an increased Firmicutes/Bacteroidetes ratio in comparison with lean individuals (Grigor'eva, 2020). Several bacterial species in intestinal diseases, for instance, colorectal cancer, seem to preferentially inhabit either on-tumor or off-tumor sites. However, members of the phylum Firmicutes displayed a disparate distribution. Some species were enriched in the on-tumor tissue, whereas others inhabited the adjacent healthy mucosa in the off-tumor sites. This demonstrated that organisms, even though belonging to the same taxonomic clade, could have different functional roles in an ecosystem. That depended on their interactions between the bacteria and their environment (Tjalsma et al., 2012). (4) Martin et al. used healthy Chilean fecal samples through the V3–V4 region of the 16S rRNA gene of bacterial DNA. They found that Verrucomicrobia (8.5 ± 10.4%) was the third most dominant strain in the gut, followed by Firmicutes (43.6 ± 9.2%) and Bacteroidetes (41.6 ± 13.1%). Besides, the microbiota of the Chilean subjects was rich in Verrucomicrobia. What is more, the mucus-degrading bacterium *Akkermansia muciniphila* was the only identified member of the Firmicutes phylum. This microorganism was a hallmark of the healthy gut due to its anti-inflammatory and immunostimulant properties and its ability to improve gut barrier function, insulin sensitivity, and endotoxinemia (Fujio-Vejar et al., 2017).

## Slow Transit Constipation in Clinical Perspective

**Figure 1:**
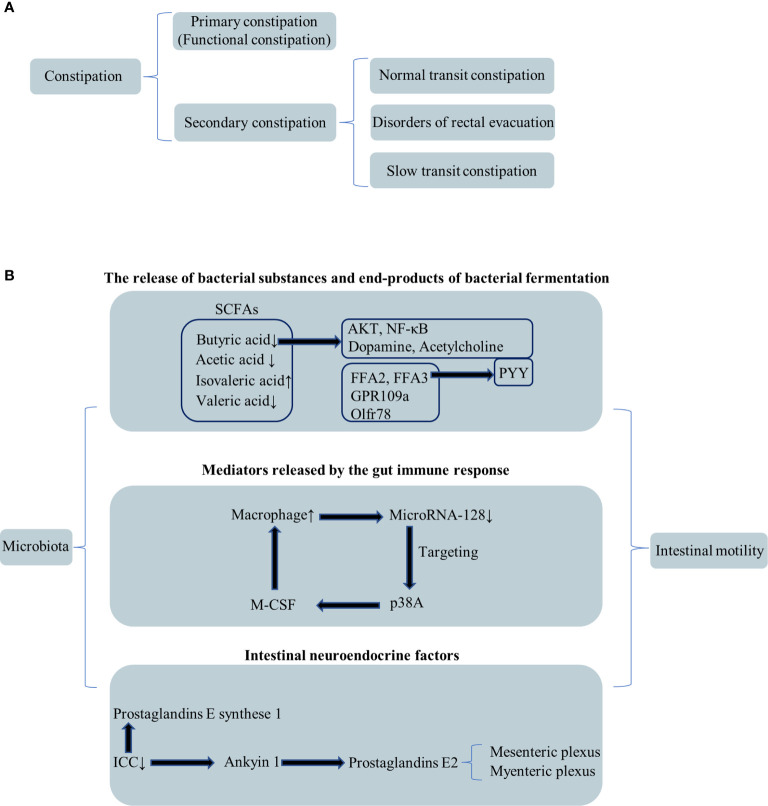
Classification of constipation and relationship among the microbiota and intestinal motility. **(A)** Secondary classification of constipation. **(B)** The relationship between the microbiota and intestinal motility through three perspectives.

Chronic constipation severely compromises the quality of life for long-term duration (Irvine et al., 2002). Constipation includes two types: primary constipation, also called functional constipation, and secondary constipation (**Figure 1A**) (Tillou and Poylin, 2017). Slow transit constipation (STC) accounts for a significant proportion of constipation cases. STC diagnosis requires evidence of slowed colonic transit. The Rome criteria, which was the most recent iteration (Rome III), was produced in 2006, providing a standardized definition of constipation (Tillou and Poylin, 2017). Patients with functional constipation were classified in three subgroups: normal transit constipation, disorders of defecatory or rectal evacuation (outlet obstruction), and STC (**Figure 1A**) (Lembo and Camilleri, 2003; Prather, 2004). Primary constipation is frequently due to constipation-predominant IBS, STC, obstructed defecation such as paradoxical contraction, pudendal neuropathy, increased perineal descent, or nonrelaxation of the puborectalis. The following six conditions contributed to the development of secondary constipation: (1) dehydration, (2) poor dietary fiber intake, (3) a variety of medications, (4) numerous medical conditions, (5) low physical activity levels, and (6) mechanical obstruction such as rectal stricture (Tillou and Poylin, 2017). While a variety of medical therapies exist, these are often met with limited success, and many patients have to face the surgery. Subtotal colectomy with ileorectal anastomosis is the most commonly performed surgical procedure (Tillou and Poylin, 2017). Nevertheless, for many patients, the surgery clinical effect is limited. The neuroendocrine system and the autonomic and enteric nervous systems have all been implicated in colonic dysmotility (Tillou and Poylin, 2017). However, the underlying mechanism of colonic dysmotility that leads to STC is ill defined.

## The Clinical Therapeutic Effects and Mechanisms of Fecal Microbiota Transplantation in Slow Transit Constipation

Li Ning’s team is an international authority in clinical trials in FMT treating STC. Recently, there have been seven published clinical trial reports about the FMT clinical applications for STC in Li Ning’s team (**Table 1-1**). In 2016, they pressed 23 STC patients who received FMT combined with soluble dietary fiber and probiotics [registered in ClinicalTrials.gov (NCT02016469)]. During the researchers’ team follow-up, the STC patient clinical improvement reached 69.6% (16/23), and the remission reached 52.2% (12/23). In the whole clinical research, no serious adverse events were observed (Ge et al., 2016a). As soluble dietary fiber and probiotics are known to have an independent effect on colon transit time, the study design has imperfection.

**Table 1-1:** The clinical efficacy of FMT reported in the literatures.

Therapy	Clinical improvement rate	Clinical remission rate	Serious adverse events	Patients number	Reference
**FMT+soluble dietary fiber+probiotics**	**69.9%**	**52.2%**	**0**	**23**	**(** Ge et al., 2016a **)**
**FMT+soluble dietary fiber**	**66.7%**	**42.9%**	**0**	**21**	**(** Ge et al., 2016b **)**
**FMT+soluble dietary fiber**	**75.9%**	**69.0%**	**0**	**29**	**(** Zhang et al., 2018 **)**
**FMT**	**50%**	**37.5%**	**0**	**24**	**(** Tian et al., 2016 **)**
**FMT**	**53.3%**	**36.7%**	**0**	**30**	**(** Tian et al., 2017 **)**

At the same year, they came out that 21 patients with STC, who received FMT on three consecutive days and soluble dietary fiber for 4 weeks (8 g, twice daily), are responding well to treatment. The clinical improvement reached 66.7%, and remission of constipated reached 42.9% (Ge et al., 2016b). In Ning Li et al.’s clinical trial, 60 patients were randomly assigned to two groups on average. Thirty patients were randomized to conventional treatment alone and another to FMT through a nasointestinal tube. The results showed that, besides not observing any serious adverse events, the FMT group had a 30% higher cure rate for treatment of STC than conventional treatment (Tian et al., 2017). In 2018, they came off the press that the FMT has a remarkable effect in STC clinical application. Li Ning’s study shows that FMT was effective and safe for STC. They compared different therapy times and found that 3 to 4 weeks receive the best benefits (**Table 1-2**) (Ding et al., 2018). Their results, published in 2018, indicate that FMT with soluble dietary in combination fiber has both short- and long-term efficacy in treating STC (Zhang et al., 2018). Li Ning’s research group compared the three treatment routes of bacterial flora transplantation between nasojejunal tube, oral enterobacterial capsule treatment, and colonoscopy infusion (pressed in 2020). Three months after treatment, they demonstrated the clinical improvement rates: 71.1% (69/97), 53.6% (45/84), and 44.0% (11/25), respectively. The nasojejunal tube route had better clinical efficacy above all (**Table 1-3**) (Tian H. L. et al., 2020). In this prospective open-label study, 24 patients with STC were enrolled. Through nasojejunal tubes, patients received FMT on three consecutive days. After treatment, there was a 12-week follow-up. Based on clinical activity at week 12, the clinical improvement rate was 50% (12/24), and the clinical remission rate was 37.5% (9/24). In this study, significant overall improvements were seen in the gastrointestinal quality-of-life index score at weeks 1, 2, 4, 8, and 12 of follow-up compared with baseline. The improvements were accompanied by the declining colonic transit time. Except for venting (6/24), abdominal pain (3/24), bloating (2/24), and diarrhea, there were no severe adverse events during the whole FMT procedure follow-up. This study, published in 2016 by Li Ning’s team, demonstrated that FMT was safe and had the potential to improve symptoms in patients with STC (Tian et al., 2016).

**Table 1-2:** The clinical efficacy of FMT during different therapy times.

Therapy time (week)	Primary efficacy endpoint achieved rate	Reference
**3-4**	**50.0%**	**(** Ding et al., 2018 **)**
**9-12**	**38.5%**	**(** Ding et al., 2018 **)**
**21-24**	**32.7%**	**(** Ding et al., 2018 **)**

**Table 1-3:** The clinical efficacy of FMT reported in different treatment routes.

Treatment route	Clinical improvementrate	Reference
**Nasojejunal tube group**	**74.2%**	**(** Tian H. L. et al., 2020 **)**
**Oral capsule group**	**60.0%**	**(** Tian H. L. et al., 2020 **)**
**Colonoscopy group**	**53.3%**	**(** Tian H. L. et al., 2020 **)**

STC and constipation-predominant irritable bowel syndrome (IBS-C) have similar clinical symptoms. Long-term follow-up results showed the IBS-C patients’ stool frequency up to 2.68 ± 1.15 times per week from 1.5 ± 1.38 times after FMT treatment within 1 month (Cui et al., 2021). In response to FMT, there was no sex difference (El-Salhy et al., 2021). Although no serious adverse events were detected, one-third of the patients had mild adverse reactions during follow-up, such as abdominal distension, nausea, vomiting, headache, and fever (Cui et al., 2021). Alexander C. Ford and his research team *via* meta-analysis demonstrated that there was no benefit according to the IBS subtype from FMT, although they grouped patients with irritable bowel syndrome with diarrhea (IBS-D), irritable bowel syndrome with mixed (IBS-M), and IBS-C together. Since only 20 patients with IBS-C enrolled, a meaningful estimate of efficacy in IBS-C could not be provided (Ianiro et al., 2019). The conflicting data of FMT in IBS-C suggested the limitation of FMT in clinical applications. It opens up new ideas for future research on pathological differences and intestinal microflora characteristics of STC and IBS-C.

Using a mice animal model to determine the mechanism underlying delayed gut motility, researchers found that short-chain fatty acids (SCFAs) and secondary bile acids were decreased in mice, which received microbiota from constipated donors (Ge et al., 2017). Compared to the STC patients, the gut microbiota compositional changes in species richness and α diversity were much higher than those in healthy volunteers. Alterations of the microbiome *via* altered microbial-derived metabolites could affect gut motility in the development of constipation, and the clinical phenotype can improve after the restoration of disturbed microbiota. That means for STC, regulating the intestinal environment will be a novel therapy strategy (Ge et al., 2017).

However, the exact molecular mechanism of FMT’s treatment of STC has not been found. The pathogenesis of STC remains largely unknown; it is a motility disorder characterized by markedly increased total bowel transit time measured by radioactive markers with a normal radiologically assessed bowel diameter (Wang, 2015). Intestinal movement disorders cause STC. The gut microbiota and bacterial fermentation products may play a prominent role in intestinal dysmotility (Ikee et al., 2020). Barbara et al. came up with a hypothesis that there are three mechanisms responsible for the effects of microbiota on intestinal motility: (1) the release of bacterial substances or end products of bacterial fermentation; (2) intestinal neuroendocrine factors; and (3) mediators released by the gut immune response (**Figure 1B**) (Barbara et al., 2005).

## Fatty Acids, Lipid Metabolism, and Metabolites

The alterations in fatty acid and lipid metabolism might be the reason causing functional constipation (Mancabelli et al., 2017). According to the number of carbon atoms in the carbon chain, the organic fatty acids with less than six carbon atoms are called short-chain fatty acids (SCFAs), which mainly include acetic acid, propionic acid, isobutyric acid, butyric acid, isovaleric acid, and valeric acid. SCFAs might aggravate the symptoms of STC *via* enhancing colonic fluid and sodium absorption. Besides, the acetate concentration in the STC patient group was significantly reduced compared with the controls (Tian H. et al., 2020). In the human gut, SCFAs are generated by bacterial fermentation sensed by specific membrane-bound receptors: FFA2, FFA3, GPR109a, and Olfr78 (Priyadarshini et al., 2018). Researchers got the consensus that the shorter the carbon chain length of the SCFA, the higher the potency at FFA2 and vice versa for FFA3 (Hudson et al., 2012).

In STC mouse stools, Qiulan He et al. found that butyric and valeric acid declined while isovaleric acid content increased. By regulating AKT and NF-κB (nuclear factor-kappaB) signaling, butyrate could improve defecation and intestinal mobility (He et al., 2020). After supplementation with butyrate and deoxycholic acid, some symptoms in mice from STC donors were reversed (Ge et al., 2017). Above all, the decrease of butyrate was closely related to the formation of STC. The SCFA receptors FFA2 and FFA3 affect peptide YY(PYY) secretion, and PYY lowers the intestinal transit rate (Beglinger and Degen, 2006).

The effect of probiotics in the gut is also achieved through short-chain fatty acids. A significant association between dysregulation of gut flora and STC has been identified, suggesting that probiotics may be an important potential treatment option for constipation in the future. Hiroshi Ohno’s team found that *Bifidobacterium bifidum* G9-1 (BBG9-1) has an effect on loperamide-induced STC in the rat model. BBG9-1 improved constipation parameters (fecal quantity, fecal water content, and fecal hardness) in constipated rats. It was found that BBG9-1 improved intestinal flora imbalance, prevented the decrease in intestinal butyric acid concentration, increased serum serotonin, and inhibited the increase in serum dopamine and the decrease in acetylcholine. In addition, increased expression of tryptophan hydroxylase 1 (5-HT synthase) was observed. These results suggested that BBG9-1 ameliorates ecological dysregulation, which leads to an increase in organic acids, further increasing intestinal fluidity, and ultimately to the relief of constipation. Therefore, the probiotics BBG9-1 might be a potential treatment option for STC (Makizaki et al., 2021).

## Intestinal Neuron

Enteric neuron could regulate gut motility by the microbiota. Vassilis et al.’s study showed that the transcription factor aryl hydrocarbon receptor (AHR) acts as a biosensor linking the intestinal environment to intestinal motility programs of the enteric nervous system (ENS) (Hindson, 2020). The transcriptional profiling in this study demonstrated that in colonic neurons, microbiota-dependent induction regulates AHR signaling or enteric excitability specific genes (Obata et al., 2020). The mice with deletion of the *AHR* gene had a longer intestinal transit time than the control mice. Enteric-neuron-specific deletion of the *AHR* gene reduced colonic peristaltic activity.

Thus, modulating AHR signaling could have potential application for the treatment of STC. In Gu et al.’s study, there were three groups of patients: healthy control group, normal transit constipation group, and STC group. Compared with the healthy control group, concentrations of neurotensin and motilin were both significantly reduced in the normal transit constipation group and the STC group. They found that the miR-19a level was the highest, whereas the circORC2 level was the lowest in the slow transit constipation group. Their study established a relationship web among circORC2, miR-19a, and neurotensin/motilin. Those indicated that the overexpression of circORC2 could upregulate the levels of neurotensin and motilin exerting a beneficial treatment for STC (Wang et al., 2021).

## Immune System

Liu et al. found a significant increase in the number of macrophages in 20 (80%) of the 25 colonic specimens from patients with STC compared with patients without a history of constipation, which was associated with downregulating the expression of microRNA-128. They further demonstrated that microRNA-128 might directly target p38A in intestinal epithelial cells to regulate the expression of the macrophage colony stimulating factor (M-CSF). Researchers hypothesized that more macrophages were recruited to the colon to activate the immune response, leading to the onset of STC. In addition, in specimens from patients with STC, the authors observed a reduction in the number of interstitial cells of Cajal (ICCs) in colon, further supporting that ICC is the marker of intestinal peristalsis (Liu et al., 2015). Treg is specialized by T cells noted for immune-suppressive effects against autoantigens. SCFAs’ receptor FFA2 could affect immune homeostasis through the regulation of the number and function of Tregs (Sakaguchi et al., 2008). Besides, SCFAs regulated their expansion in the intestine through FFA2 signaling (Priyadarshini et al., 2018).

## Interstitial Cells of Cajal and Others

Other investigations found that the quantitative alternations of the ICC are the significant biomarker of the gastrointestinal tract motility. Researchers used antibodies of CD117 or DOG1 as the ICC’s biomarker. They demonstrated a complete absence or significant reduction in the number of ICC in colon specimens resected from patients with STC compared with normal controls (He et al., 2000; Geramizadeh et al., 2009). Prostaglandins have been reported to play an indispensable role in the mechanism of intestinal motility (Staumont et al., 1988; Dey et al., 2006) through regulating the function of intestinal smooth muscle (Tokita et al., 2015). Prostaglandin E_2_ acted on individual myocytes and coordinated peristalsis *via* the mesenteric and myenteric plexus (Haupt et al., 2000). Mesenchymal cells promoted colorectal contraction by two ways: (1) synthesizing the vital enzymes needed in prostaglandin synthesis, such as prostaglandin E synthase 1, and (2) expressing transient receptor potential ankyrin 1 to activating Prostaglandin E_2_ release (Yang et al., 2019). Lubiprostone, a Prostaglandin E_2_ derivative, alleviated small bowel bacterial overgrowth in patients with chronic constipation (Sarosiek et al., 2016) and modulated the pacemaker activity of ICC in mice (Jiao et al., 2014). Above all, the researchers’ findings demonstrate the dynamic interaction between the gut microbiome, epithelial function, and intestinal motility.

## Future Perspective for FMT

FMT is a promising treatment at present. However, only one randomized controlled trial has been performed yet; more evidence is needed to certify FMT as an available clinical therapy for STC. Furthermore, FMT to be considered as a first choice is unlikely due to the challenges in identifying donors as well as the complexity of the procedure. FMT might be more suitable in patients who are refractory to conventional therapeutic strategies. In most studies, donors were recruited without checking the microbial profile (Ohkusa et al., 2019). It is not nearly enough to say what one person’s gut microbiota does to another. In addition to safety issues, the lack of standards of behavior is currently a barrier to the spread of FMT. Strict implementation standards are still incomplete, and systematic data on the efficacy and long-term and short-term adverse reactions of FMT are still lacking, including the need to determine and monitor the improvement or deterioration of each patient’s response to FMT. With the uncertain pathogenesis and the complexity of microorganisms in the human gut, there is a long way to unraveling the veil of STC pathogenesis and the mechanism of FMT in STC. FMT is the first and most natural method to change the intestinal microecology, especially in the treatment of STC, which highlights the effects that both doctors and patients agree on. However, in clinical practice, the treatment effect declines over time, and repetitive FMT is required to acquire a sustained effect. Besides, its long-term prognosis and overall advantages and disadvantages are not fully understood. In the future, more animal and clinical trials will be needed to further validate this treatment. Even if the current evidence is not sufficient, we still have high expectations and full confidence about the future therapeutic effects of FMT.

## Author Contributions

JL, LG, YL, and XZ designed the paper, contributed to manuscript writing, and approved the manuscript before submission. JL and LG collected literatures and approved the manuscript before submission. SZ, MZ, and MW gave constructive comments on the manuscript and approved the manuscript before submission. All authors contributed to the article and approved the submitted version.

## Funding

This work was supported by grants from the Foundation of the committee on science and technology of Tianjin (20JCQNJC01870) and Joint Scientific Research Project between Nankai University and Tianjin Union Medical Center (2016rmnk002).

## Conflict of Interest

The authors declare that the research was conducted in the absence of any commercial or financial relationships that could be construed as a potential conflict of interest.

## Publisher’s Note

All claims expressed in this article are solely those of the authors and do not necessarily represent those of their affiliated organizations, or those of the publisher, the editors and the reviewers. Any product that may be evaluated in this article, or claim that may be made by its manufacturer, is not guaranteed or endorsed by the publisher.

## Abbreviations

AHR, aryl hydrocarbon receptor; BBG9-1, *Bifidobacterium bifidum* G9-1; CDI, *Clostridium difficile* infection; ENS, enteric nervous system; FMT, Fecal microbiota transplantation; IBD, Inflammatory bowel disease; IBS, Irritable bowel syndrome; IBS-C, constipation-predominant irritable bowel syndrome; IBS-D, irritable bowel syndrome with diarrhea; IBS-M, irritable bowel syndrome with mixed; ICC, interstitial cells of Cajal; M-CSF, macrophage colony stimulating factor; NF-κB, nuclear factor-kappaB; PYY, peptide YY; SCFA, Short-chain fatty acid; STC, Slow transit constipation.
